# Comparing pharmaceutical pricing and reimbursement policies in Croatia to the European Union Member States

**DOI:** 10.3325/cmj.2011.52.183

**Published:** 2011-04

**Authors:** Sabine Vogler, Claudia Habl, Martina Bogut, Luka Vončina

**Affiliations:** 1 Health Economics Department, WHO Collaborating Centre for Pharmaceutical Pricing and Reimbursement Policies, Gesundheit Österreich GmbH / Geschäftsbereich ÖBIG – Austrian Health Institute, Vienna, Austria; 2 Department for Drugs and Medical Products, Croatian Institute for Health Insurance, Zagreb, Croatia

## Abstract

**Aim:**

To perform a comparative analysis of the pharmaceutical pricing and reimbursement systems in Croatia and the 27 European Union (EU) Member States.

**Methods:**

Knowledge about the pharmaceutical systems in Croatia and the 27 EU Member States was acquired by literature review and primary research with stakeholders.

**Results:**

Pharmaceutical prices are controlled at all levels in Croatia, which is also the case in 21 EU Member States. Like many EU countries, Croatia also applies external price referencing, ie, compares prices with other countries. While the wholesale remuneration by a statutorily regulated linear mark-up is applied in Croatia and in several EU countries, the pharmacy compensation for dispensing reimbursable medicines in the form of a flat rate service fee in Croatia is rare among EU countries, which usually apply a linear or regressive pharmacy mark-up scheme. Like in most EU countries, the Croatian Social Insurance reimburses specific medicines at 100%, whereas patients are charged co-payments for other reimbursable medicines. Criteria for reimbursement include the medicine’s importance from the public health perspective, its therapeutic value, and relative effectiveness. In Croatia and in many EU Member States, reimbursement is based on a reference price system.

**Conclusion:**

The Croatian pharmaceutical system is similar to those in the EU Member States. Key policies, like external price referencing and reference price systems, which have increasingly been introduced in EU countries are also applied in Croatia and serve the same purpose: to ensure access to medicines while containing public pharmaceutical expenditure.

Since 1990, the Croatian health care system has changed considerably. Reforms were undertaken to totally re-organize the health care system by introducing privatization of primary health care and institutional changes to increase efficiency and ensure accessibility and funding for health care (1-5). In 1999, the Croatian Institute for Health Insurance (CIHI), as key third-party payer, was set up (6), as well as another institution, the Agency for Medicinal Products and Medical Devices, which started to work in 2003 (7). At that time, Croatia, which had been granted candidate status for the European Union (EU) in June 2004 (8), had already been working for years on harmonizing its pharmaceutical legislation with the “*acquis communautaire*” (ie, accumulated legislation, legal acts, and court decisions, which constitute the body of European Union law) (7). This concerned, in particular, the regulatory framework regarding marketing authorization that is harmonized in the EU (9). Further major components of a pharmaceutical system, besides market authorization, are pharmacovigilance, pricing, reimbursement, and distribution; for all of which specific regulation is in place in most countries with a well-developed health system. In the EU, pharmaceutical pricing and reimbursement remains the competence of the Member States. While all EU Member States have to comply with the rules of the Transparency Directive (10) which aims at guaranteeing pricing and reimbursement decisions to be taken in a transparent way within specific time-frames, it is up to the country how they shape their pharmaceutical pricing and reimbursement system.

As part of their obligation to the fulfillment of the right to health, states have the obligation to grant access to essential medicines, ie, medicines that fulfill the priority needs of their population (11-13).

In many countries the world over, this is done by the provision of a rather limited range of medicines in public sector facilities that are procured by the state. While eligible patients can access essential medicines in the public sector either free of charge or with a modest co-payment, they have to purchase out-of-pocket medicines in the private sector (14-17).

Europe has higher health service coverage, ie, reimbursement of health expenditure by a social health insurance or a national health service, compared with the rest of the world. This is reflected in the share of the public funding of health and pharmaceutical expenditure. In the EU, around 75% of health expenditure and two thirds of pharmaceutical expenditure is on average covered by the public payers (18). All EU countries have been struggling with the rise in pharmaceutical, in particular publicly funded, expenditure (Table 1).

**Table 1:** Development of pharmaceutical expenditure in Croatia and in the European Union Member States, 2000-2005 and 2000-2008 (or latest available year)*

	Index of pharmaceutical expenditure in			
Country	2000-2008 (index year 2000 = 100)		2005-2008 (index year 2005 = 100)	
	total	public	total	public
Austria (AT)	156	156	120	125
Belgium (BE)	n.a.	n.a.	113	124
Croatia (HR)	n.a.	152^†^	n.a.	109^†^
Cyprus (CY)	n.a.	n.a.	127^‡^	109^‡^
Czech Republic (CZ)	228	183	119	97
Denmark (DK)	150^‡^	172^‡^	112^‡^	112^‡^
Finland (FI)	152^‡^	172^‡^	103^‡^	108^‡^
France (FR)	149	148	111	107
Germany (DE)	138	146	110	114
Greece (EL)	268^‡^	336^‡^	136^‡^	147^‡^
Hungary (HU)	n.a.	n.a.	111	97
Ireland (IE)	303	353	136	144
Italy (IT)	123	131	101	96
Latvia (LV)	212	248^§^	156	122^§^
Lithuania (LT)	279^‡^	n.a.	142^‡^	n.a.
Luxembourg (LU)	139^║^	142^║^	n.a.	n.a.
Malta (MT)	131^‡^	220^‡^	105^‡^	122^‡^
Netherlands (NL)	154	153	116	116
Poland (PL)	n.a.	n.a.	135	136
Portugal (PT)	139^§^	138^§^	103^§^	100^§^
Slovakia (SK)	254	220	134	130
Slovenia (SI)	n.a.	n.a.	116	112
Spain (ES)	207	206	119	121
Sweden (SE)	134	111	110	106
United Kingdom (UK)	116	125	96	97

*Source: OECD Health Data 2010 (19), supplemented by data validation and data provision from PPRI network members (national statistics for Croatia, Cyprus, Finland, Lithuania, Latvia, Malta, Netherlands). Data are missing for Bulgaria, Estonia, and Romania (n.a.).

†Social insurance expenditure on prescription medicines.

‡2000-2007 and 2005-2007, respectively.

§2000-2006 and 2005-2006, respectively.

║2000-2005.

Cost-containment is also an issue for high-income countries. They apply a range of supply-side and demand-side measures, both direct and indirect, targeting the price and/or the volume components. In the field of pricing and reimbursement, different frameworks for the regulation of prices and distribution margins are in place and different methodological approaches and tools are applied to assess which prices are considered acceptable for the public payer as well as for the individual. Most EU countries opted for price regulation for, at least, reimbursable medicines and regulation of wholesale and, in particular, pharmacy margins (18,20-32). Countries have been elaborating criteria (eg, cost-effectiveness, relative effectiveness, medical need) for deciding when and how much reimbursement should be granted. Health economics, which is also referred to as “fourth hurdle” (33), has been increasingly used in European reimbursement decisions (34-36). With the institutionalization of health economics in pharmaceutical reimbursement, health technology assessment (HTA) has been gaining importance as a basis for decision-making (37). Already observable during the last decade, but reinforced by more recent literature (32,38,39), the tools of external price referencing (international price comparisons) and reference price systems (limitation of reimbursement for identical or similar medicines in a cluster) have been increasingly used in several EU Member States. Comparing the evidence for the application of different policy options, numerous changes could be observed that addressed the introduction or abolition of a measure as a whole as well, and also rather often, the way the instrument is designed. Each country has its own individual approach to framing its pharmaceutical pricing and reimbursement system for reacting to the challenges, and the way how they do this is influenced by culture and tradition (18).

In 2009 and 2010, Croatia substantially reformed its regulation on pharmaceutical pricing and reimbursement. A maximization of “value for money” was one of the major objectives strived for in the reform (40).

This article presents the current pharmaceutical pricing and reimbursement system in Croatia following the reform and explores its similarities and differences compared with other European countries. Since Croatia is candidate for the EU membership, the 27 EU Member States were taken as reference. The scope of harmonization to the *acquis communitaire* from a regulatory framework is not addressed in this study.

## Materials and methods

### Indicators for benchmarking pharmaceutical systems

Pharmaceutical pricing and reimbursement is one key area where regulators and public payers apply a bundle of policy measures to reconcile the goals of providing the population with effective and new medicines and of meeting budgetary limits.

Analyses in both peer-reviewed and gray literature (20-32,38,39) have been focusing on specific pharmaceutical pricing and reimbursement regulations and policy measures, which have been in place in numerous European countries.

We will survey these key “standard” policy measures for pharmaceutical pricing and reimbursement. As methodological framework we have chosen the Pharmaceutical Health Information System (PHIS) indicators (41), which can be considered as the most recent indicators for pharmaceutical systems from a public health perspective in Europe. The PHIS indicators build on experience in this field since they were developed based on earlier indicators projects, in particular on three European Commission commissioned projects: EURO-MED-STAT (42), SOGETI indicators (43), and Pharmaceutical Pricing and Reimbursement Information (PPRI) (18,44).

For the comparative analysis of pharmaceutical pricing and reimbursement in this article, we selected the following PHIS (sub)indicators:

• with regard to pricing:

− price control at manufacturer level,

− key pricing policies at manufacturer level,

− external price referencing,

− price control at distribution level (wholesale, pharmacy).

• with regard to reimbursement:

− reimbursement eligibility,

− reimbursement lists (positive lists, negative lists),

− reimbursement rates,

− reference price system,

− co-payments.

Even if health and pharmaceutical expenditure have been defined among the three core indicators of the total of the 23 PHIS indicators, expenditure data will not be presented in the Results section, which aims to focus on policy options. Data about pharmaceutical expenditure’s growth in European countries were provided as background information in the Introduction section.

While the PHIS indicators’ methodology framework covers both the outpatient and inpatient sector, we address only the outpatient sector.

### Information on the Croatian pharmaceutical system

A review of international literature was carried out by searching the Internet in general and, in particular, the PubMed database and Google Scholar. Search terms included “pharmaceutical policy,” “medicines,” “pharmaceutical,” “pricing,” “reimbursement,” “Croatia,” “Europe,” “EU,” alone and in combination with each other. The review focused on studies published in English, but also considered documents in German. Furthermore, gray literature was taken in consideration, as well reports and materials recommended by interview partners. Furthermore, the bibliography of articles and reports was checked for other relevant studies.

In addition, we contacted the CIHI, which is the national competent authority for pricing and reimbursement of medicines, and the Croatian Association for Pharmacists. In personal interviews with experts of the CIHI in autumn 2009 important data were gained and compiled in a factsheet with information as of end of 2009 (45). In summer 2010, follow-up contacts with CIHI staff were made for clarification, validation of information, and for providing updates. Two authors from CIHI contributed to this paper as co-authors and are thus guarantors of correct current data on Croatia. The information on Croatia presented in this study is as of July 2010.

### Information on the pharmaceutical systems in the 27 EU Member States

Information about the pharmaceutical pricing and reimbursement systems in the EU Member States has been gathered for years and is available in the WHO Collaborating Centre for Pharmaceutical Pricing and Reimbursement Policies, established at the Health Economics Department of the Austrian Health Institute (Gesundheit Österreich GmbH), to which two of the authors are affiliated. However, we needed a specific update for this article.

In 2007/2008, the authors affiliated to Gesundheit Österreich did a primary research on key pharmaceutical pricing and reimbursement information. This was done in the course of the PPRI project, at that time an EU-funded project. We collected the information and data through country reports, the so-called PPRI Pharma Profiles (25),  which were written according to an outline around major indicators by country experts, coming mainly from national public authorities in the field of pharmaceutical pricing and reimbursement (the PPRI network members). Data for the comparative analysis were validated and/or added by the PPRI members.

The data in this “pharmaceutical pricing and reimbursement information system” established by the Health Economics Department of the Austrian Health Institute (Gesundheit Österreich GmbH) have been regularly updated.

One reason for constant monitoring is that the institute operates on a legal basis (46) the Pharma Price Information (PPI) service (47) about pharmaceutical prices in all EU Member States since the institute is mandated to check prices submitted by manufacturers to the Austrian Pricing Committee (Austria applies external price referencing). For interpreting the prices correctly, we have to know and understand the underlying regulatory framework.

In order to present the most updated information and data in this article, we did an extra validation, and we address the PPRI network members. Even if PPRI as an EU-funded project came to its end in 2008, the PPRI network continues as a Member States-driven initiative and has even enlarged. It now covers national public pricing and reimbursement agencies in 37, still mainly European, countries. The collection of most up-to-date information for this study consisted of two surveys. First, for the PPRI network meeting in November 2009, the PPRI participants were asked to outline in a poster their current pharmaceutical pricing and reimbursement system according to the key indicators, which have been applied for this article, and the expected reforms as of 2010. Those countries that had announced major changes for 2010 were followed up in e-mail and telephone correspondence in the first months of 2010. Second, to be sure that we did not miss any reforms, we launched a so-called PPRI query in September 2010, asking in a structured format for policy changes (major measures were listed) of the first two quarters of 2010 and expected reforms for the rest of the year 2010. In addition of these two surveys, extra correspondence and conversations were needed for clarifications as well as for checking on phone with participants who had not responded. The list of PPRI network members is shown in Table 2.

**Table 2:** Institutions participating in the Pharmaceutical Pricing and Reimbursement Information (PPRI) network*

Country	Institution
Austria (AT)	Gesundheit Österreich GmbH / Geschäftsbereich ÖBIG – Austrian Health Institute (GÖG/ÖBIG) – PPRI Secretariat Austrian Federal Ministry of Health (BMG) Main Association of Austrian Social Security Institutions Austrian Chamber of Labour
Belgium (BE)	Health Insurance Institute
Bulgaria (BG)	International Healthcare and Health Insurance Institute
Croatia (HR)	Croatian Institute for Health Insurance
Cyprus (CY)	Health Insurance Organization Ministry of Health
Czech Republic (CZ)	Ministry of Health Medicines Agency Charles University
Denmark (DK)	Medicines Agency Ministry of Interior and Health
Estonia (EE)	Ministry of Social Affairs
Finland (FI)	Ministry of Social Affairs and Health
France (FR)	National Sickness Fund for Employees Ministry of Health, Youth and Sport University Claude Bernard Lyon 1
Germany (DE)	Institute for Medical Documentation and Information Ministry of Health
Greece (EL)	Institute for Pharmaceutical Research and Technology National Organization for Medicine
Hungary (HU)	National Health Insurance Fund
Ireland (IE)	Health Service Executive - Finance Shared Service National Centre for Pharmacoeconomics
Italy (IT)	Medicines Agency
Latvia (LV)	Centre of Health Economics
Lithuania (LT)	Ministry of Health
Luxemburg (LU)	Ministry of Health Union of Sickness Funds
Malta (MT)	Ministry of Social Policy, Health, the Elderly and Community
Netherlands (NL)	Ministry of Health, Welfare and Sport
Poland (PL)	Ministry of Health
Portugal (PT)	Medicines Agency (INFARMED)
Romania (RO)	Ministry of Health
Slovakia (SK)	Medicines Agency Ministry of Health
Slovenia (SI)	Agency for Medicinal Products and Medical Devices
Spain (ES)	Ministry of Health and Social Policy Andalusian School of Public Health
Sweden (SE)	Pharmaceutical Benefit Board
United Kingdom (UK)	Medicines Pharmacy and Industry, Department of Health

*With regard to the focus of this article, only institutions from Croatia and the EU Member States are listed. Additionally, 9 further countries and European and international institutions are also part of the PPRI network (see *http://ppri.goeg.at*).

To guarantee consistency with the time-frame of the information collected for Croatia, we present the information about the EU Member States as of July 2010.

### Terminology

This article is consistent with the PHIS Glossary (48), which is a major terminology resource on pharmaceutical policies.

## Results

### Pricing at manufacturer level

In most EU Member States, manufacturer prices are directly regulated by the state. However, in a few EU countries (Cyprus – for imported medicines, Denmark, Finland, Latvia, the Netherlands, Poland, Sweden, and United Kingdom) the manufacturer price is indirectly regulated, ie, with the maximum wholesale price being approved by the authorities in the Nordic countries or via the Pharmaceutical Pricing Regulation Scheme (PPRS) scheme controlling the maximum profit of companies in the United Kingdom (32). Croatia controls the wholesale price and also indirectly the ex-factory price due to a statutorily regulated wholesale mark-up of maximum 8.5% on the ex-factory price.

At manufacturer level, Croatia and most EU Member States control pharmaceutical prices only for reimbursable medicines. Only 5 EU countries apply price regulation to all medicines (Table 3).

**Table 3:** Pricing policies and procedures at manufacturer level for medicines in Croatia and in the European Union (EU) Member States, 2010*

Pricing policies/procedure	Croatia and EU Member States
**Price control at manufacturer level:**	
for all medicines	BE, CZ, EL, LU, LV^†^
for reimbursable medicines	AT, DE^‡^, DK^†^, EE, ES, FI, FR, HU, **HR**, IE, IT, LT, PL, SE, SI, SK, UK^§^
for prescription-only medicines	BG, NL^†^, PT, RO
others	CY^║^, MT^¶^
**Key pricing policies:**	
statutory pricing	AT, BE, BG, CY, CZ, EE, EL, ES, FI, **HR**, LT, NL, PT, RO, SE, SI, SK
price negotiations	FR, IT
other policy	DE**, DK**, MT^††^, UK^‡‡^
mix of policies	LV^§§^, HU^║║^, IE^¶¶^, PL^§§^
**External price referencing:**	
in place	AT, BE, BG, CY, CZ, EE, EL, ES, FI***, FR, **HR,** HU, IE, IT^†††^, LT, LU, LV, NL, PL, PT, RO, SI, SK
basket: <6 countries	CY, EE, FR, **HR,** LT, LU, NL, PT, SI
basket: 6-12 countries	BG, IE
basket: >12 countries	AT, BE, CZ, EL, ES, FI***, HU, LV, PL, RO, SK

*Source: Survey by the authors, information provided by members of the Pharmaceutical Pricing and Reimbursement Information network. Country abbreviations are explained in the Table 1.

†At wholesale level.

‡Reimbursable off-patent products.

§National Health Service medicines.

║Locally produced medicines.

¶Public sector.

**Basically free pricing.

††Tendering (public sector).

‡‡Indirect price control through profit control based on Pharmaceutical Pricing Regulation Scheme.

§§Statutory pricing after price negotiations.

║║Price negotiations take place in addition to statutory pricing criteria.

¶¶The “statutory” basis is an agreement, in case of non-availability of data price negotiations take place.

***No formal price comparison, but prices of other EU Member States must considered.

†††Number of reference countries not specified.

In Europe, Denmark and Germany (not taking into consideration the reforms starting to be implemented in 2011) are considered as the two most liberal, ie, free-pricing countries (49,50). Nonetheless, there is a strong linkage between pricing and reimbursement in several systems (51), and this is also true for these two countries. In the reimbursement market, there is free pricing for on-patent medicines, but price control is applied for off-patent products.

The most common pricing policy is statutory pricing, where medicine prices are set on a legal basis (eg, law, enactment, decree) (48). This is the case in Croatia (Pricing Decree, Official Gazette No. 155/09) and in a number of other European countries. Price negotiations as single policy measure are rare; however, they are sometimes used in combination with statutory pricing (Table 3). Quite often, statutory pricing is followed by a negotiation process between the public payer and the pharmaceutical company when it comes to reimbursement of medicines.

A common pricing procedure in the EU countries is external price referencing (international price comparison). External price referencing is also applied in Croatia. The basket of countries serving as reference for Croatia comprises Slovenia, Italy, France, and sometimes Spain and the Czech Republic (40,45). External price referencing is applied in 22 EU Member States and usually the basket of reference countries is quite small (18,30,32).

Specific pricing policies often apply for generics, whose price in the reimbursement market is set at a certain percentage below the price of the original product (52). In Croatia, the first generic available will have the price set 30% below the originator and each subsequent generic will be 10% below the previous generic on the Croatian positive list (29).

### Pricing at distribution level

Pricing at distribution level refers to the remuneration of distributors (wholesalers and retailers) for their services of handling, distributing, and dispensing medicines.

In Croatia, a statutory maximum wholesale mark-up of 8.5% of the ex-factory price is applied (45). Seven EU Member States also opted for regulating the wholesale remuneration via a linear add-on, whereas 14 countries apply a regressive mark-up scheme for wholesale (Table 4). In Cyprus (only for locally-produced medicines), Denmark, Finland, the Netherlands, Sweden, and the United Kingdom, no statutory wholesale mark-up is in place at all (53).

**Table 4:** Statutory wholesale and pharmacy remuneration in Croatia and in the European Union (EU) Member States, 2010*

	Statutory wholesale and pharmacy remuneration in Croatia and EU Member States	
	wholesale	pharmacy
**Scope:**		
for all medicines	AT^†^, BE, CZ, EE, EL, ES, **HR**, HU, LU, LV, PT, SI^‡^	AT^§^, BE, CY – private sector, CZ, DK^║^, EE, EL, ES, FI^¶^, **HR**, HU, LU, LV, PT, SI^‡^
for reimbursable	DE**, FR, IE^††^, IT, LT, PL, SK	DE**, FR, IE^††^, IT, LT, PL, SK, UK
for prescription-only medicines	BG, RO	BG, NL, RO, SE
for others	CY^‡‡^, MT^§§^	MT^§§^
**Form:**		
linear	CY, EL, **HR**, IE^††^, IT, MT, PL, PT	CY, DK, EL, IT^║║^, MT, PT
regressive	AT, BE, BG, CZ, DE, EE, ES, FR, HU, LT, LV^¶¶^, RO, SI, SK	AT, BE, BG, CZ, EE, ES, FI, FR, HU, LT, LV^¶¶^, PL, RO, SE***, SK
fees	-	**HR**, NL, SI, UK
others	LU^†††^	DE^‡‡‡^, IE^††^, LU^†††^

*Source: Survey by the authors, information provided by members of the Pharmaceutical Pricing and Reimbursement Information network. Country abbreviations are explained in the Table 1.

†Two different schemes depending on reimbursement category.

‡Except non-reimbursable over-the-counter.

§Two different schemes for different kind of customers.

║Except over-the-counter sold outside the pharmacy.

¶Except nicotine replacement therapy sold outside the pharmacy.

**Two different schemes for prescription-only medicines and for reimbursable over-the-counter.

††For different reimbursement schemes.

‡‡For locally produced medicines.

§§For medicines in the private sector.

║║Made regressive by mandatory pharmacy discounts.

¶¶Different schemes for reimbursable and for non-reimbursable medicines.

***Plus fee for generics.

†††Linear and regressive schemes depending on the origin of the product.

‡‡‡Flat fee plus linear mark-up for prescription-only medicines, regressive for reimbursable over-the-counter.

Regarding the pharmacy sector, all EU Member States have implemented a statutory remuneration scheme. The most common one is the remuneration by a regressive mark-up scheme, but a few countries have decided for the policy option of offering (dispensing) fees or charges for the services which a pharmacy performs (Table 4). Such a fee-for-service remuneration can be found in Croatia, the Netherlands, Slovenia, and the United Kingdom. In Croatia, the pharmacy service fee is calculated on granting specific points for different services (eg, dispensing, accounting activities, preparation of antibiotics of oral use) (45).

Pharmacists in Croatia are allowed to substitute a prescribed medicine with a product of the same or cheaper price if the prescribed one is not on the market. However, the reference price system, which is in place in Croatia, might motivate patients to ask for the least expensive alternative, as the CIHI always pays the reference price.

### Reimbursement eligibility

In Croatia, the competent authority for reimbursement is the CIHI, which acts as major third party payer for medicines. Having been granted a marketing authorization, a pharmaceutical company may apply for reimbursement for its product at CIHI. In the reimbursement decision, the Reimbursement Committee acts as an advisory body that, following an evaluation of the application, recommends based on specified criteria if a medicine is eligible for reimbursement and on which of the two Croatian reimbursement lists it should be placed. The final decision is taken by the board of the CIHI (45).

Such an approach is called product-specific reimbursement (48), meaning that the third party payer (either a social health insurance institution or a national health service) decides about the reimbursement eligibility of a specific medicine. This product-specific approach is also applied in 19 of the 27 EU Member States (eg, Belgium, Czech Republic, Greece, Finland, Italy, the Netherlands, Poland, the United Kingdom) (18,30,32).

The reimbursement eligibility of medicines could also be linked to diseases (eg, in the Baltic states, where the same medicine may be reimbursed at different rates depending on the indication) or to population groups, which is the key reimbursement eligibility scheme in Cyprus, Ireland, and Malta. In Denmark and Sweden, reimbursement coverage increases with rising pharmaceutical consumption of a patient (expressed in her/his pharmaceutical expenditure within a year), thus asking the patient to pay 100% of her/his medication at the beginning and offering (nearly) full reimbursement after a certain out-of-pocket spending threshold has passed – this is called consumption-based reimbursement eligibility. In the EU countries, the key scheme, which is usually product-specific eligibility, might be supplemented by another scheme (eg, population group-specific reimbursement, since higher reimbursement rates might be in place for vulnerable groups).

### Reimbursement lists and reimbursement criteria

If in Croatia a medicine is considered eligible for reimbursement, it will be put on one of the two positive lists:

• List A, which is the basic list providing 100% reimbursement of the reference price for listed products (eg, Clexane, Amlopin, Simvastatin) or

• List B, where patients are charged co-payments (eg, Fosamax, Voltaren).

All EU countries have reimbursement lists. Positive lists, which include medicines that may be prescribed at the expense of a third party payer, are very common and are in place in 23 EU Member States (all but Germany, Greece, Spain, and the United Kingdom). Four countries (Germany, Hungary, Spain, and the United Kingdom) have negative lists which explicitly exclude medicines from reimbursement (18,30,32,53). Greece has provided a legal basis for negative lists, but has not implemented this measure yet. Indeed, Greece is currently considering the re-introduction of the positive list, which had been abolished in 2006 (Table 5).

**Table 5:** Reimbursement lists and rates and out-of pocket payments in Croatia and in the European Union (EU) Member States, 2010*

Reimbursement	Croatia and EU Member States
**Reimbursement lists:**	
positive list(s)	AT, BE, BG, CY, CZ, DK, EE, FI, FR, **HR**, HU, IE, IT, LT, LU, LV, MT, NL, PL, PT, RO, SE, SI, SK
negative list(s)	DE, EL^†^, ES, HU, UK
**Reimbursement rates:**	
only 100%	AT, DE, IE, IT, MT, NL, UK
100% and further rates	BE, BG, CY, CZ^‡^, DK, EE, EL, ES, FI, FR, **HR**, HU, LT, LU, LV, PL, PT, RO, SE, SI, SK
**Co-payments/out-of-pocket payments (multiple types of co-payments possible):**	
fixed fee (eg, prescription fee)	AT, DK, EE, FI, FR, **HR,** HU, IT^§^, PL, SK, UK
percentage co-payment	BE, BG, CY, CZ, DE^║^, DK^¶^, EE, EL, ES, FI, FR, **HR,** HU, LT, LU, LV, PL, PT, RO, SE^¶^, SI, SK
other out-of-pocket payments	IE**, MT^††^

*Source: Survey by the authors, information provided by members of the Pharmaceutical Pricing and Reimbursement Information network. Country abbreviations are explained in the Table 1.

†There is a legal basis, but the negative list has not been implemented yet. Currently, the re-introduction of positive list is ongoing.

‡No fixed rates defined except for bacterial/immune stimulants.

§In some regions.

║It is a prescription fee of 10% of the medicine’s price (with absolute minimum and maximum).

¶Depending on pharmaceutical expenses of the patient.

**Out-of pocket payments after a certain threshold of pharmaceutical expenses under a specific reimbursement scheme.

††In the private sector.

Being included in the positive list does not automatically mean that the cost of the medicine will be fully covered by the third party payer. Like Croatia, many EU countries grant 100% reimbursement for selected medicines (eg, usually essential and life-saving medicines), while other reimbursable medicines are reimbursed at lower rates, eg, at 75%, 50%, 40%, and 20% in Belgium (54) or at 69%, 37%, and 15% in Portugal (55,56). Only in 7 EU Member States are all medicines eligible for reimbursement fully reimbursed (Table 5).

In most European countries, the criteria for the decision on the inclusion of a medicine into reimbursement and the reimbursement rates are health-economic parameter. Usually the cost-effectiveness of a medicine, the medical need, the therapeutic value, in particular the relative effectiveness (in case of medicines with no new, but added therapeutic value), and – increasingly, the budget impact (eg, in the Central and Eastern European countries) – are assessed (18,25,34-36).

In Croatia, a key criterion for inclusion into reimbursement is clinical effectiveness, which is defined as allows: importance of the medicine from a public health perspective, its therapeutic importance and its relative therapeutic value, ethical aspects and its quality, the reliability of data, and assessment from reference sources. There is no formal HTA assessment in Croatia’s reimbursement system. All reimbursement applications for original medicines must contain a budget impact analysis in line with the ISPOR guidelines (40).

### Reference price system

Croatia has a reference price system, as do 20 EU Member States (Table 6). A reference price system is a pharmaceutical reimbursement element in which identical or similar products are clustered in so-called reference groups. For each cluster, a maximum amount (reference price) to be covered by the third party payer is decided. The patient must pay the difference between this reference price and the actual pharmacy retail price of the medicine, in addition to any other co-payments.

**Table 6:** Reference price system in Croatia and in the European Union (EU) Member States, 2010*

Reference price systems	Croatia and EU Member States
**Existence:**	
in place	BE, BG, CZ, DE, DK, EE, EL, ES, FI, FR, **HR**, HU, IT, LT, LV, NL, PL, PT, RO, SI, SK
abolished	SE
never introduced	AT, CY, IE, LU, MT, UK
**Clustering of reference groups:**	
at ATC 5 level	BE, DK, EE, EL, ES, FI, FR, IT, LT, PT, RO, SI
at ATC 5 and 4 level	BG^†^, CZ, HU^†^, SK
at ATC 5, 4 and 3 level and at different criteria	DE, **HR**, LV, NL, PL
**Calculation of reference price:**	
lowest price of products in the group	BG, CZ, DE, DK, EE, FR, HU, **HR**, IT, LT, LV, PL, RO, SI, SK
below average of prices of products in the group	DE, ES, FI
(around) average	(BE^‡^), EL, NL
above average	PT

*Source: Survey by the authors, information provided by members of the Pharmaceutical Pricing and Reimbursement Information network. Country abbreviations are explained in the Table 1.

†Anatomic Therapeutic Chemical classification 4 (ATC, *http://www.whocc.no/atc/structure_and_principles/*) only for a few products.

‡The reference price is set at 30% below the price of the original product in the cluster, which eventually leads to a reference price around the average of the prices of all products in the reference group, as the setting of the reference price is regularly followed by price decreases undertaken by manufacturers of the products in the group.

In Croatia, the clustering is done based on a broad definition of a reference group, taking into consideration identical and similar products at ATC 5, 4, and 3 level (45). Most EU countries apply a rather strict understanding of a cluster, which is built on products with the same active ingredient (ATC 5) and even the same pharmaceutical form (57,58).

Croatia decided to take as the reference product the lowest priced product that accounts for at least 5% of the sales in the cluster, which is in line with the procedure chosen by the majority of the EU countries. Fifteen EU Member States reimburse at the level of the lowest price as well, while the remaining countries define the maximum reimbursement amount around and above the average of the prices of medicines in the group (Table 6).

The reference price system is regularly updated in Croatia. Frequent adaption to changes is also the case in many EU countries. Most of them update on a quarterly level, some even on a monthly level, and Denmark updates every two weeks (59).

### Co-payments

Patients have to pay out of pocket for pharmaceutical expenditure that is not covered by the state. This concerns private expenses for self-medication, but also any kinds of co-payments.

In Croatia, co-payments for medicines on the positive list B are applied. Additionally, a prescription fee of HRK 15 (around € 2) per prescription is charged for reimbursable medicines. Finally, due to the reference price system, co-payments may also occur if the patient opts for a product on List B with a price above the reference price (45).

Percentage co-payments are also the most common co-payment in the EU countries (21 EU Member States), as most countries apply different reimbursement rates for the products on the positive list. Prescription fees are in place in 10 EU countries (Table 5). No co-payments at all (apart from those applicable under the reference price system) are charged in some regions in Italy, in the public sector in Malta, and in the Netherlands (18,30,32,51,59,60).

Usually, EU Member States have mechanisms for vulnerable groups (eg, low income people, people with chronic diseases, etc.), which might take the form of lower reimbursement rates or exemptions from co-payments (61). In Croatia, specific medicines (in particular some orphan medicines) are excluded from co-payments, since the government runs a special budget for these medicines. The practice of having specific budgets for orphan medicines or cost-intensive medicines, usually used in hospital settings, is also known from a few EU Member States (eg, France, the Netherlands) (62).

## Discussion

This study offers updated information on key features of the pharmaceutical pricing and reimbursement systems of 28 European countries. We consider this piece of work to be of added value, since we provide not only recent information on Croatia, whose pharmaceutical sector has been reformed substantially during the last two years, but also a complete and updated picture of all EU Member States. Evidence, in particular peer-reviewed literature, is often limited to a few EU countries, mostly large Western European countries. There have been in-depth overviews and analyses of pharmaceutical pricing and reimbursement regulation in several EU countries around the millennium (20,21,23,24,26), but these pieces of information have become out-dated following reforms which countries have undertaken, and the EU has meanwhile been enlarged by 12 further countries. A highly appreciated exemption is some work on the generics sector which addresses the Central and Eastern European countries (28,29,58).

This study was performed for the out-patient sector. This is in line with most evidence in the literature (20,21,23,24,26), where the in-patient sector is disregarded completely or only few elements are considered. We believe that more attention should be placed on the in-patient sector since the start of a treatment with a specific medicine has a major impact on the further use in the out-patient sector. Nonetheless, in this article we addressed only the out-patient sector in order not to overload this study. We took this decision in the light of the fact that pricing and procurement policies and reimbursement strategies in the in-patient sector differ considerably from the out-patient sector (62).

**Figure 1:**
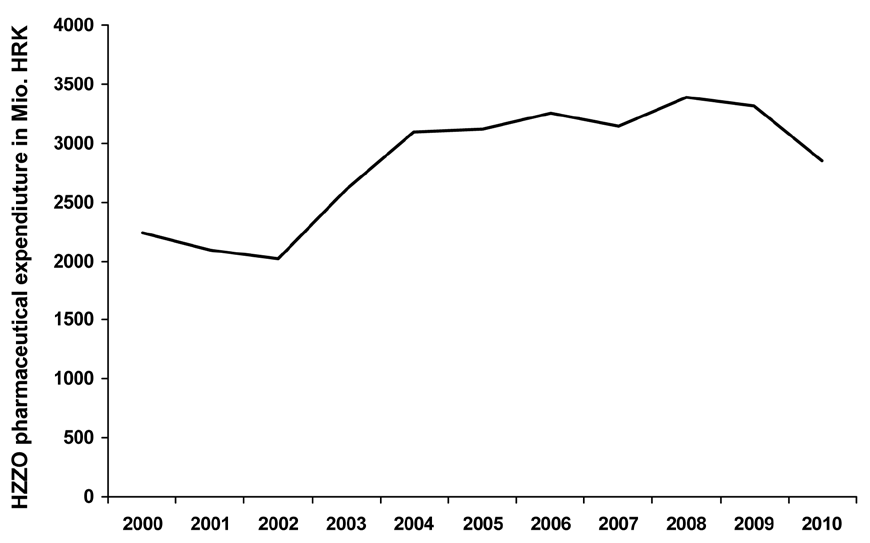
Expenditure on prescription medicines of the Croatian Institute for Health Insurance (CIHI) 2000-2010, expressed in million Croatian Kuna (€  1=  HRK 7.4), data provided by the CIHI.

Why is it of such relevance that the information is updated? Croatia has been implementing a set of policy measures to get better “value for money,” ie, to be able to grant best medication to the population within given financial limits. While between 2002 and 2008 an increase in CIHI expenditure for prescription medicines was observed for each year, Croatia succeeded in generating some savings in 2009 and 2010 (Figure 1).

At the same time, EU Member States have also been struggling with growth rates in (public) pharmaceutical expenditure (Table 1). Cost-containment has been a major motivator for policy reforms in pharmaceutical pricing and reimbursement in the EU Member States (20,21,23). The growth in (public) pharmaceutical expenditure demands that countries react regularly, since measures that had been successful in short term lose its effectiveness due to the “pendulum effect” (23) (ie, after some time, usually two to three years, players have learned the rules of the games and make use of loopholes in the system). Besides striving for cost-containment, some EU Member States have adapted their regulation of pharmaceutical pricing and reimbursement systems to comply with the European Commission’s legal requirements (ie, Transparency Directive). In its recent reform, Croatia, while having the focus on the pharmaceutical bill, also introduced criteria for more transparency and accountability (eg, introduction of a court instead of an arbitration procedure for appeal of the Social Insurance’s reimbursement decision) (40). This appears to be a good preparation for future action within the EU regulatory framework.

On the pricing side, Croatia applies price regulation for reimbursable medicines at manufacturer level, as do many EU countries. A pricing procedure which has been increasingly applied for regulating prices is external price regulation (international price comparison). The updated information in the article shows that today this procedure is used in 22 countries, some of which introduced it in the course of the last 10-15 years (18,23,32).

European countries have increasingly opted for implementing this tool in spite of criticism. International price comparisons are considered extremely sensitive to choices made about certain key methodological issues, such as sample selection, unit of measurement for price and volume, the relative weight given to consumption patterns in the countries being compared, and the use of exchange rates or purchasing power parities for currency conversion (63,64). It has been suggested that prices should be weighted by consumption when being used for international price comparison. However, this is not the case in any European country. It would probably entail a lot of administrative work including high costs for acquiring the consumption data. Another concern about external price referencing is that it is considered as “readily game-able by the pharmaceutical industry” (38) and might contribute – by reducing pharmaceutical companies’ willingness to price to market – to access and affordability problems in lower-income countries due to launch delays (65). Given the provided information and the design of this study, we cannot assess if this has been or will be problem for Croatia. In any case, we have not been made aware of such problems in Croatia yet.

An analysis (18) of external price referencing in Europe revealed that countries tend to focus on a rather small basket of reference countries, and they tend to chose neighboring countries, countries with which they have historical links and economic relationships and – in particular if they themselves rank among middle- or low-income countries – countries with a more or less low pharmaceutical price level. Some countries have a rather well-defined methodology in the legal framework, while others allow some flexibility. Analyzing the methodology applied for external price referencing, similarities of the Croatian system to the EU countries were identified: Croatia has a quite small basket of reference countries, however allowing the inclusion of a few further countries, and it focuses on low-price countries. The current framework of external price referencing in Croatia appears to offer a greater potential for savings.

In the field of distribution, the Croatian regulatory framework is partly similar to the EU countries, with regulated maximum allowed remuneration for both wholesalers and pharmacies. To remunerate pharmacies, Croatia opted for a performance-based system (fee-for-service remuneration), which is rather rare in EU countries, only seen in Croatia’s neighboring country Slovenia and – to some extent – in the Netherlands and UK. Whereas a pharmacy’s remuneration, organized as a margin scheme, is based on the prices of the medicines dispensed, a fee for service remuneration takes the broader range of pharmacy services into account. While regulated mark-ups and margins continue to be the major form of regulating pharmacy remuneration in Europe, performance-based remuneration might be one option which EU countries could consider and implement when re-organizing the pharmacy sector.

Regarding reimbursement, health economics, considered as “fourth hurdle” (33), plays an increasingly important role in Europe (34-36). This is also reflected in the current reimbursement legislation in Croatia in which, for instance, a budget impact analysis is asked for. A key element in the Croatian reimbursement system is a reference price system, which has been introduced in more and more countries (66). While referencing pricing was identified for only 5 EU Member States (out of the EU-15 Member States at that time) a decade ago (21), the present update shows that it is in place in 20 EU Member States (thereof 10 countries which were EU Member States at the time of EU-15). The rather late introduction of a reference price system in some EU countries can be attributed to the fact that for building clusters in the reference price system a sufficient number of generics has to be on the market (67). In several, in particular Western European, countries it took some years until, due to the expiry of patents, this critical number became available, while Central and Eastern European countries, including Croatia, have always had a stronger generics market (18,29). There is evidence that the existence of rather broad clusters combined with low reference prices contributes to optimizing savings (59). In fact, Croatia applies a broad definition of a reference group whereas most EU countries opted for sticking to a rather strict scope of a cluster.

A reference price system is usually seen as one policy option within the bundle of measures for generic promotion (68,69), since it is a tool, if well designed, for developing and promoting a generics market (70). A study (18) provided evidence that in Europe the instruments of generic substitution and reference price system usually go hand in hand because these two tools influence each other positively. Croatia also opted for this approach and implemented both tools. Nonetheless, generic substitution in Croatia is neither mandatory nor motivated by a financial incentive that could serve as a positive factor for increasing generic uptake (59). However, in Croatia incentives for generic promotion are not considered necessary since the Social Insurance pays the reference prices, and as a consequence most manufacturers lower their prices to avoid co-payments.

This paper presents an analysis at macro level, ie, based on benchmarks for describing a pharmaceutical pricing and reimbursement system. It might be limited in its conclusions, since for performing a comparison of nearly 30 countries we needed to compromise in some cases and simplify the presentation of data in order to achieve clarity. The study does not provide answers on the success and failure of specific policy measures implemented in Croatia. There are indications of a positive impact of the recent pharmaceutical pricing and reimbursement reform on public pharmaceutical expenditure, but the middle- and long-term effects on expenditure, and also on accessibility and affordability for the patients can be only be assessed in the coming years. Such an evaluation should consider the whole spectrum of the Croatian pricing and reimbursement reform. Besides the policies described in this study and included in the comparison, the reform covered further important elements: improvements in decision-making and transparency have been made, eg, applications for the inclusion of products are now published on the Web site. Importantly, Croatia has been addressing the sensitive issue of expensive medicines and provided for a separate budget for these expensive medicines and for a payback agreement mechanism between the manufacturers and the Social Insurance (40,45).

Croatia has been undertaking reforms in the pharmaceutical system for more than 10 years. Apart from changes in the marketing authorization procedure (6) and in pricing and reimbursement, reforms have been made to shape the system for ensuring a more rational use of medicines. In the last years, a focus has been placed on improving physicians’ prescribing pattern, which was considered as extensive. This was addressed by awareness-raising projects and performance indicators in the contracts (71,72). A major current issue in Croatian pharmaceutical policy concerns promotional activities by pharmaceutical companies. In the 2009/2010 reform, a uniform agreement on the ethical promotion of medicines was introduced on a mandatory basis, and its implementation is guaranteed by a financial revolving deposit mechanism where pharmaceutical companies are obliged to deposit their promotional budgets to the Social Insurance (40).

The comparison in this article did not tackle all aspects of a pharmaceutical system.

Our comparative exercise is a starting point for assessing where Croatia stands regarding its pharmaceutical pricing and reimbursement framework in Europe and which countries with a similar organization of the pharmaceutical system Croatia could address for exchanging information and experiences about policy options.

This scene-setting might serve as a good basis for an in-depth analysis that looks more closely at some policy measures and combines indicators. For instance, the fact that a country has a positive list is a preliminary indicator of access to medicines, however the number and the kind of medicines selected to be included in the positive list and the reimbursement rates have also be taken into consideration in a more detailed assessment (73). The PHIS indicators proved to be an adequate instrument for this comparative analysis; however, the reimbursement criteria (eg, role of health economics) should be more highlighted and could be defined as an indicator of its own.

### Conclusion

Each health and pharmaceutical system has its own country-specific characteristics which have been established due to historical developments, traditions, and culture. As a result, there are 27 pharmaceutical pricing and reimbursement systems in the EU.

Even if the individual organization of the pharmaceutical system differs in its details among the countries, some patterns and trends can be observed. In the EU Member States, external price referencing as a procedure for setting the price of medicines and reference price systems for limiting the reimbursement amount have become increasingly used instruments. Both policy measures are also in place in Croatia, since Croatia has to address the same challenge as the EU countries, namely fostering prompt, affordable access to the latest effective medicines within financial restraints. For successfully containing public pharmaceutical expenditure in the long run, countries have to adapt their policies continuously. Indeed, EU Member States are in the process of re-shaping their pharmaceutical policy frameworks on a regular basis. Croatia has recently launched a major reform which has led to savings in pharmaceutical expenditure.

It is recommended that Croatia closely monitor and evaluate the effects of the newly introduced policy measures on the development of pharmaceutical expenditure at the expense of the Social Insurance and the patients in order to assess the impact of the reform on accessibility and affordability. Some further areas should be looked at. We identified as major research issues to be addressed the volume component (data on the consumption of medicines, policy options for promoting a more rational prescribing and use of medicines) as well as the hospital pharmaceutical sector with its impact on overall pharmaceutical expenditure and the functioning of the interface management.

Croatia has developed a modern pharmaceutical pricing and reimbursement system which appears to be able to react to the challenges of patients’ needs within given financial limits. The way the system is organized is similar and comparable to the countries of the EU. This allows Croatia’s authorities and institutions to actively participate in European networks. Croatian authorities’ staff is encouraged to continue sharing their experiences on policy measures with their colleagues in other countries, since such networking is a fruitful exercise allowing all involved parties to learn lessons from each other.
